# Concomitant glenohumeral injuries in Neer type II distal clavicle fractures

**DOI:** 10.1186/s12891-018-1944-7

**Published:** 2018-01-19

**Authors:** Tobias Helfen, Georg Siebenbürger, Florian Haasters, Wolfgang Böcker, Ben Ockert

**Affiliations:** 1 Department General, Trauma and Reconstructive Surgery, Ludwig Maximilians University, Munich University Hospital, Nußbaumstr. 20, 80336 Munich, Germany; 2 Department Knee, Hip and Shoulder Surgery, Schön Klinik Munich-Harlaching, Harlachinger Str. 51, 54817 Munich, Germany

**Keywords:** Distal clavicle fracture, Concomitant injuries, Arthroscopic treatment, Osteosynthesis

## Abstract

**Background:**

To identify the prevalence of concomitant glenohumeral injuries in surgically treated Neer type II distal clavicle fractures and relate its clinical importance.

**Methods:**

Between 11/2011 and 11/2015 41 patients, suffering from a displaced and unstable distal clavicle fracture were included. 20 patients (group 1) received surgical treatment by means of plate osteosynthesis in combination with an arthroscopically assisted coraco-clavicular ligament augmentation. In group 2 (*n* = 21 patients) the fracture was treated by hooked plating solely, and diagnostic arthroscopy was conducted during hardware retrieval after the fracture had healed. All arthroscopies were performed in a standardized fashion, images were blinded retrospectively, and evaluated by two independent investigators.

**Results:**

In total, concomitant glenohumeral pathologies were found in 26.8% of cases (41 patients, mean age 43.6 ± 16.6 years). In Group 1 (*n* = 20, arthroscopically assisted fracture treatment) the prevalence was 25%, in Group 2 (*n* = 21, diagnostic arthroscopy during implant removal) 28.5% (*p* = 0.75). Concomitant glenohumeral injuries included Labrum- and SLAP-tears, partial and full thickness rotator cuff tears as well as lesions to the biceps pulley system. Concomitant injuries were addressed in 2 patients of group 1 (10%, 2× labrum repair) and in 3 patients of group 2 (14.3%, of Group 2 (2× arthroscopic cuff repair of full thickness tear, 1× subpectoral biceps tenodesis in an type IV SLAP lesion, *p* = 0.68).

**Conclusion:**

The present study could clarify the acute and for the first time mid-term implication and clinical relevance of concomitant glenohumeral injuries. They have been observed in averaged 27% of Neer type II distal clavicle fractures at these two times. However, the findings of this study show that not all concomitant lesions remain symptomatic. While lesions are still present after fracture healing, it’s treatment may be depicted upon symptoms at the time of implant removal. In turn, early diagnosis and treatment of concomitant injuries seems reasonable, as untreated injuries can remain symptomatic for more than 6 months after the fracture and recovery may be delayed.

## Background

Fractures of the distal clavicle account for approximately 17% of all clavicle fractures [1]. For stable and non-displaced fractures conservative treatment leads to satisfactory outcome, however in unstable fractures surgical treatment is recommended [2]. Surgical treatment consists of closed or open reduction followed by fracture fixation, nevertheless, numerous surgical techniques have been described including k-wire, plating, hookplating or suture fixation.

Recently, arthroscopic assisted treatment of displaced distal clavicle fractures has been reported with the advantage of a minimal invasive approach, early recovery and good functional outcome. However, as arthroscopic assisted treatment is increasingly performed concomitant intraarticular glenohumeral pathologies are observed with distal clavicle fractures [3]. Most commonly, injuries involve the superior labrum anterior-posterior complex (SLAP), the biceps pulley or the rotator cuff and account for approximately 25–46% of cases.

Associated glenohumeral injuries have been described in acute distal clavicle fracture, as well as in dislocation of the acromioclavicular joint, however, the implication and clinical relevance in the treatment of distal clavicle fractures is yet unknown [4–6]. Purpose of this retrospective study was to evaluate the prevalence of concomitant injuries in surgically treated displaced distal clavicle fractures. Furthermore, glenohumeral lesions should be evaluated for clinical relevance. We hypothesised that the prevalence of concomitant injuries is similar when assessed during diagnostic arthroscopy of successfully healed distal clavicle fractures and would represent a pathology with clinical implication.

## Methods

This retrospective study was conducted by approval of the local ethical board. Between November 2011 and November 2015, patients ≥18 years of age with a displaced fracture of the distal clavicle were included and surgically treated. Patients with a history of shoulder surgery or symptoms of glenohumeral pathology (instability, rotator-cuff tear, biceps tendon pathology) prior to the fracture event were excluded.

Fracture displacement was identified on standard radiographs in anterior-posterior as well as in Rockwood’s view and were classified as described by Neer [2]. Patients were excluded if the fracture was non displaced or other than a Neer II type. All remaining patients were subsequent divided according to the received treatment in 2 groups.

In group 1 (*n* = 20) patients received surgical treatment by means of locked plating and arthroscopically assisted coracoclavicular fixation (Clavicle Fracture System, Arthrex®, Naples, USA). Arthroscopically assisted coracoclavicular fixation consisted of a thorough diagnostic arthroscopy through a standard posterior portal including 8 steps of imaging: After entrance into the glenohumeral joint by use of blunt trocar, the first view was the triangle formed by the long biceps tendon, the humeral head and the subscapularis tendon. 2nd view was onto the biceps origin and the superior labrum anterior-posterior (SLAP) complex. The biceps origin was manipulated using a probe in order to detect a SLAP lesion. 3rd and 4th views included the medial and lateral Pulley system, the superior glenohumeral ligament (SGHL) and the encircled long biceps tendon. Hereafter the articular surface of the glenoid and humerus and the medial gleno-humeral ligament (MGHL) were examined (5th view). The 6th view was on the supraspinatus tendon and the footprint in abduction and external rotation, while the 7th view was used to exclude infraspinatus tendon tears and avulsion of the teres minor or a posterior avulsion of the glenohumeral ligament (reversed HAGL). Finally, the 8th view was to assess the axillary recess for the presence of loose bodies, lesions to the inferior gleno-humeral ligament (IGHL) and HAGL lesions. Cartilage injury of the glenoid or the humeral head was evaluated for in all of 8 standard views. Subacromial arthroscopy was not performed in patients of group 1.

Patients of group 2 (*n* = 21) received open reduction and locking plate osteosynthesis with 3.5 mm Clavicle Hook Plate (LCP, DePuy Synthes®, Zuchwil, Switzerland). All patients of group 2 underwent diagnostic arthroscopy at the time of implant removal, which was then performed in the identical manner as described above. In addition, a subacromial arthroscopy was conducted prior to implant removal.

Patients of both groups were clinically and radiographically followed for 12 months. Functional outcome has been recorded and is presented by the Constant Score (CS), the Oxford Shoulder Score (OSS) and the abduction. Arthroscopic images were blinded retrospectively in order to eliminate interpretational bias by the surgeon or the physical examination. All images were evaluated independently by two investigators. Full thickness rotator cuff tears were classified according to Bateman [7], partial supraspinatus tendon lesions were classified in accordance to Ellman [8], subscapularis tendon lesions were further classified as described by Fox and Romeo [9]. Injuries of the superior labrum in relation to the biceps tendon anchor were classified according to Snyder et al. [6] and Maffet et al. [10] Lesions of the pulley system were classified as described by Habermeyer et al. [11]

Data was enrolled through Microsoft Excel 2010 (Microsoft, Redmond, WA), followed by a statistical analysis using IBM SPSS Statistics, version 25 (SPSS, Chicago, IL). Data are given in terms of the arithmetic mean and standard deviation. The study of Pauly et al. was used to estimate the power and sample Size [12]. The authors reported the prevalence of concomitant intra-articular pathologies following high-grade dislocation of the acromioclavicular (AC) joint with 15%. Based on these findings, a sample size of 35 is needed with a 95.0% confidence interval and a normal approximation of 0.150. Frequencies were calculated for ordinal data, and the χ^2^ test was applied for group comparison. Rational data were described by mean and standard deviation. To compare the groups, analysis of variance and post hoc tests were used for parametric data, and the Mann-Whitney *U* test was used for nonparametric data. *P* ≤ .05 was considered significant for differences in group comparison.

## Results

Of 41 patients (mean age: 43.6 ± 16.6 years) with a Neer type II displaced fracture of the distal clavicle, concomitant glenohumeral pathologies were found in 11 patients (27%).

In Group 1 (*n* = 20, mean age: 53 ± 17.5 years, fracture pattern: Neer IIa *n* = 3, Neer IIb = 17) concomitant glenohumeral pathologies were detected in 5 patients (25%) during primary arthroscopically assisted treatment (Table 1). In Group 2 (*n* = 21, mean age: 39.7 ± 14.6, *p* = 0.01, fracture pattern: Neer IIa *n* = 1, Neer IIb = 20) glenohumeral pathologies were found in 6 patients (28.5%) during diagnostic arthroscopy at the time of hardware removal 6.7 ± 3.5 months following primary non-arthroscopic treatment. Concomitant glenohumeral injuries included SLAP-lesions, SSP transmural and partial lesions, SSC partial lesions as well as Pulley and Bankart lesions. (Table 1). Distribution of classification of the injuries is shown in Table 2.

**Table 1:** Distribution and significance of concomitant injuries

Concomitant injury	Group 1[n]	Group 2[n]	*p*-value
SLAP-lesion	0 (0%)	1 (4.7%)	*1*
SSP-transmural tear	1 (5%)	2 (9.5%)	*1*
SSP-partial rupture	2 (10%)	3 (14.3%)	*1*
SSC partial rupture	0 (0%)	1 (4.7%)	*1*
Pulley lesion	0 (0%)	1 (4.7%)	*1*
Labrum lesion	2 (10%)	0 (0%)	*0.23*
Bursitis glenohumeral	0 (0%)	2 (9.5%)	*0.48*

**Table 2:** Distribution of degrees of injuries according to their individual classification

Injury classification	Group 1	Group 2
SLAP-lesion *(Snyder)*	–	N = 1 *(VI)*
SSP transmural tear *(Bateman)*	N = 1 *(II)*^a^	N = 1 *(III)*
		N = 1 *(VI)*
SSP-partial rupture *(Ellman)*	N = 2 *(I)*^a^	N = 2 *(I)*a
		N = 1 *(II)*a
SSC partial rupture *(Fox/Romeo)*	–	N = 1 *(Ib)*a
Pulley lesion *(Habermayer)*	–	N = 1 *(II)*a
Labrum lesions	N = 2	–

^a^only arthroscopic debridement

**Fig. 1:**
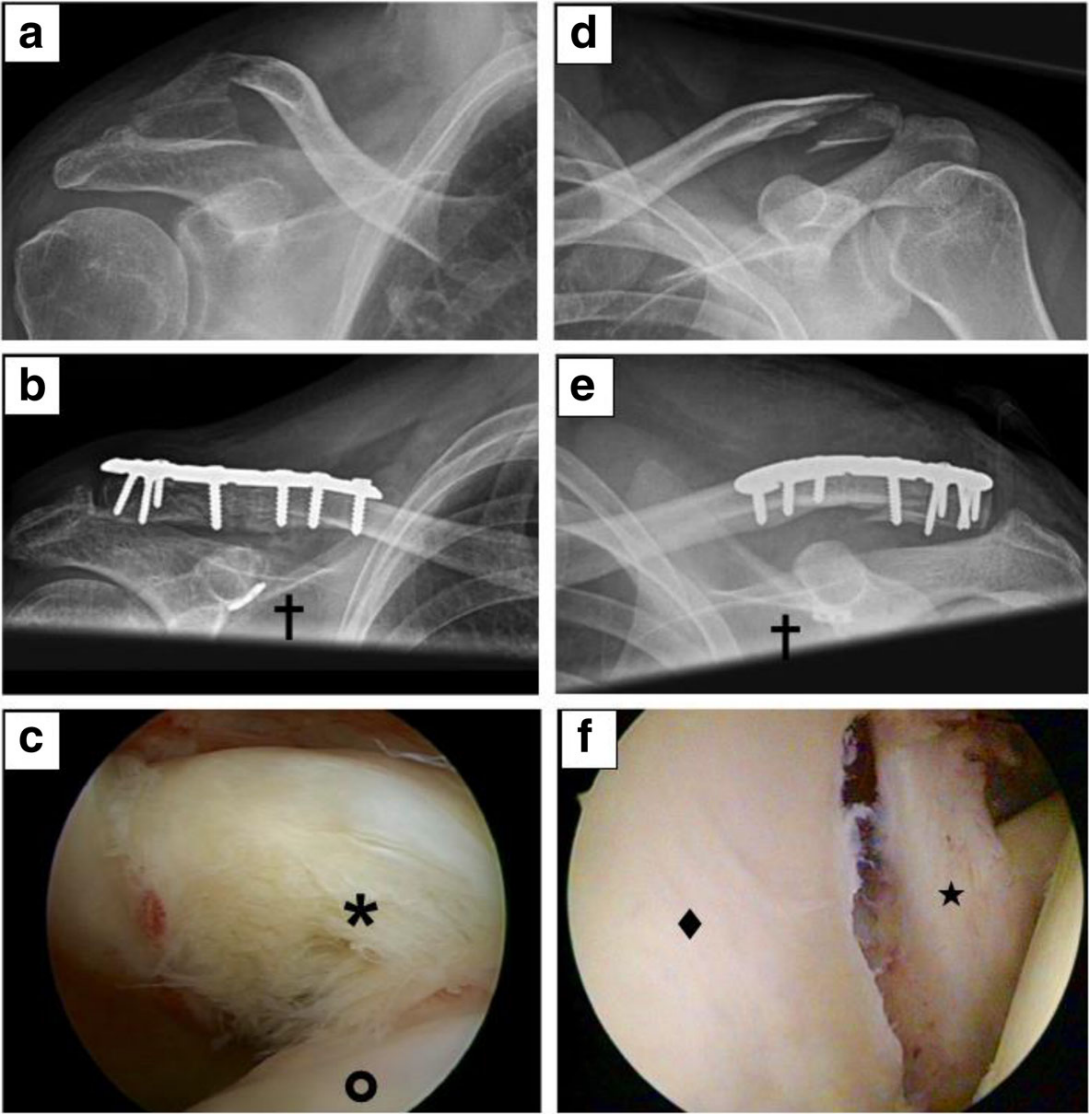
Findings of Group 1 (initial arthroscopy). **a**-**c** 53 years old female with Neer type IIb fracture treated by plate osteosynthesis with coraco-clavicular augmentation [✝]. **a** Preoperative x-ray. **b** Postoperative x-ray. **c** SSC Lesion (Fox/Romeo Ib) [✶] humeral head in front [O]. **d**-**e** 32 years old male with Neer type IIb fracture treated by plate osteosynthesis with coraco-clavicular augmentation [✝]. **d** Preoperative x-ray. **e** Postoperative x-ray. **f** anterior Bankart Lesion [★], glenoid in the front[♦]

**Fig. 2:**
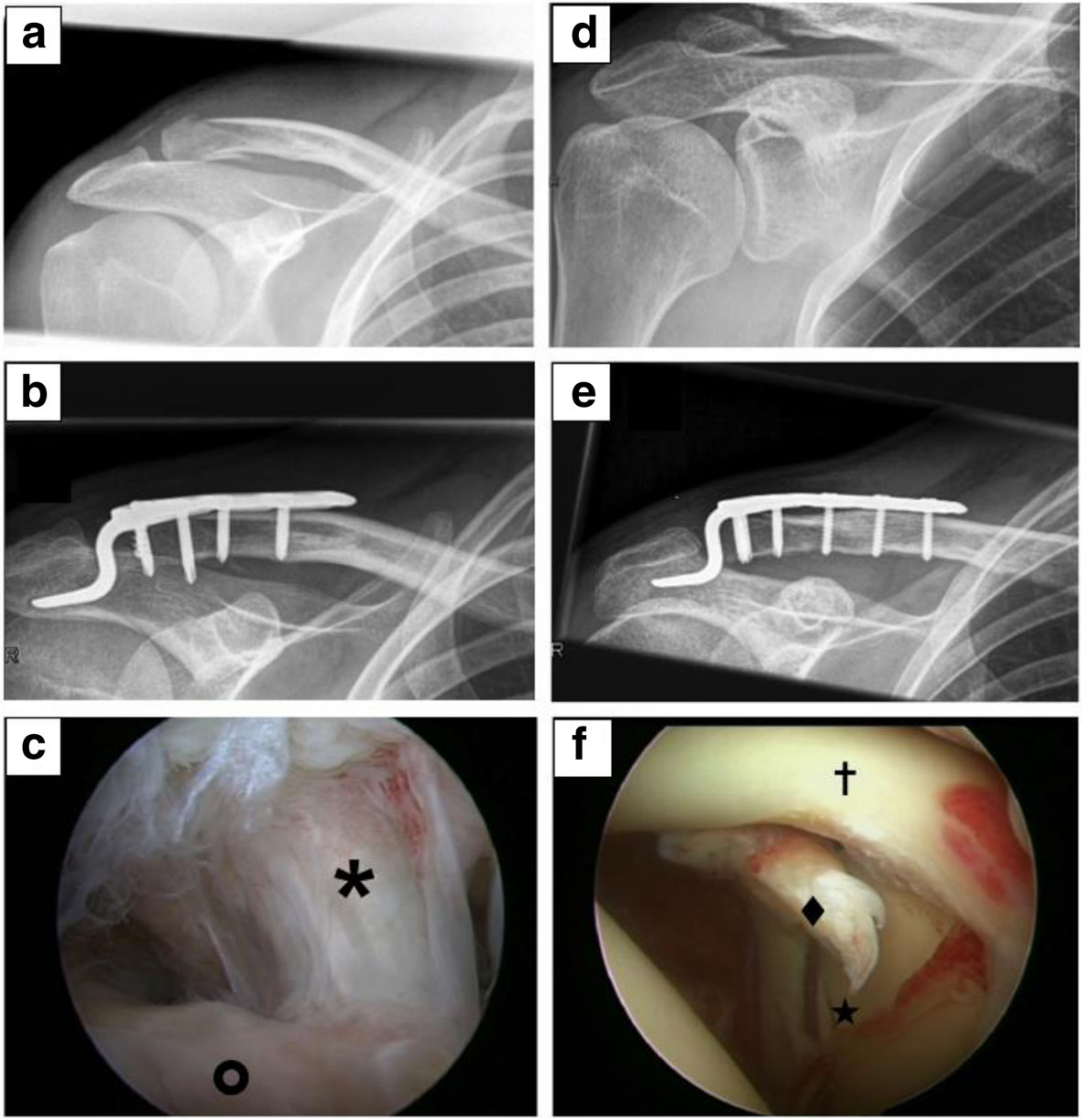
Findings of Group 2 (late arthroscopy). **a-c** 40 years old male with Neer type IIb fracture treated by hook-plate osteosynthesis. **a** Preoperative x-ray. **b** Postoperative x-ray. **c** SSP transmural Lesion (Bateman III) [✶], footprint area in front [O]. **d-e** 24 years old male with Neer type IIb fracture treated by hook-plate osteosynthesis. **d** Preoperative x-ray. **e** Postoperative x-ray. **f** SLAP Lesion (Snyder IV) [♦], long biceps tendon[✝], glenoid [★]

A distinct treatment, other than debridement, was performed in 2 patients (10%, 2 x labrum refixation) of Group 1 and in 3 patients of group 2 (14.3%, 2× arthroscopic cuff repair, 1 x subpectoral biceps tenodesis in SLAP type IV). A characteristic example for the findings is shown in Fig. 1. Of group 2, 5 patients (23.8%) patients presented with ongoing clinical symptoms related to the glenohumeral finding. 1 patient (4.8%) showed a positive Jobe’s Test (full thickness rotator cuff tear and 1 patient had a positive O’Brien’s Test (SLAP type IV lesion). The last case is exemplary presented in Fig. 2. Three patients of group 2 (14.3%) complained of persisting subacromial impingement symptoms until hook plate removal, without glenohumeral finding.

Results of the CS and the OSS as well as the degrees of abduction are shown in Tables 3 and 4. Functional outcome parameters were mainly superior in group 1. Significance was given only in the overall abduction values regardless of concomitant injuries. In the subgroup of existing concomitant injuries of group 2 implant removal and late arthroscopy benefits the patients’ functional outcome of all measured functional outcome parameters.

**Table 3:** Functional outcome

	Group 1	Group 2	*[p]*
CS total	92.2 ± 5.6	90.6 ± 8.9	*0.49*
CS (-CI)	93.6 ± 4.9	90.9 ± 9.7	*0.33*
CS (+CI)	87 ± 4.9	88.8 ± 1.9	*0.43*
OSS total	46.9 ± 2.4	45.6 ± 4.9	*0.28*
OSS (-CI)	45.5 ± 5.6	47.4 ± 2	*0.23*
OSS (+CI)	44.8 ± 3.3	43.3 ± 6.7	*0.69*
CS (late)	–	90.8 ± 1.9	
OSS (late)	–	45.3 ± 4.6	

*CS* Constant Score, *(-CI)* without concomitant injuries, *(+CI)* with concomitant injuries, *OSS* Oxford Shoulder Score, *(late)* after late arthroscopy and surgical intervention

**Table 4:** Range of Motion (Abduction)

	Group 1	Group 2	*[p]*
Abduction [°]	179 ± 3	172 ± 13	*0.02*
Abd [°] (-CI)	180 ± 2	174 ± 13	*0.06*
Abd [°] (+CI)	177 ± 4	163 ± 17	*0.15*
Abd [°] (late)	–	169 ± 9	

*(-CI)* without concomitant injuries, *(+CI)* with concomitant injuries, *(late)* after late arthroscopy and surgical intervention

## Discussion

Fractures to the distal third of the clavicle represent 10–30% of all clavicle fractures and can be treated conservatively with satisfying outcome in the majority of cases. However, symptomatic non-union under conservative treatment exists and therefore, distal clavicle fractures with instability should be treated operatively, with respect to patient’s age and functional demands. Over the last decades, surgical treatment of distal clavicle fractures developed from open reduction and fixation by k-wires, conventional plates or hook-plating to minimal invasive approaches and arthroscopically assisted fracture management. Arthroscopically assisted fracture fixation may be beneficial in terms of minimally invasive approach as well as assessment and treatment of associated glenohumeral lesions. While impaired functional outcome and prolonged pain was historically contributed to fracture non-union, several authors noted that other reasons for a limited shoulder function may be present [13]. In this context, due to an increase of arthroscopic assisted fracture treatment, concomitant glenohumeral lesions were observed more frequently and proclaimed as potentially causing shoulder dysfunction [14].

Beirer et al. detected concomitant intra-articular glenohumeral injuries in 13 of 28 patients (46.4%) with initially suspected isolated lateral clavicle fracture. Surgical treatment was performed in 8 of 28 cases (28.6%) of which superior-labrum-anterior-posterior (SLAP) lesions, injuries to the pulley-complex as well as rotator cuff tears were regarded as relevant injuries. The authors concluded that subsequent surgical treatment of these formerly missed but therapy-relevant injuries may increase functional outcome and reduce complication rate. However, several arthroscopic and imaging studies have shown that there are glenohumeral pathologies in otherwise asymptomatic patients. Tempelhof et al. showed a prevalence of rotator cuff tears in 13% of patients aged 50 to 59 years with an overall rate of 23% of all patients with asymptomatic shoulders [15].

In the present study glenohumeral injuries, concomitant to a displaced fracture of the distal clavicle were found in 27% of cases. This result supports the data of existing studies reporting of acute determined concomitant glenohumeral injuries in 25–46% of patients with distal clavicle fractures [3]. For the first time, our study showed that glenohumeral pathologies are not only observed during primary arthroscopic fracture treatment, but are also present more than 6 months thereafter. No study exists to show late arthroscopy findings in distal clavicle fractures. With the particular collective of patients undergone late arthroscopy we could examine this issue.

Some concomitant injuries could potentially heal others are clinically irrelevant. In some cases this turns out only in the further course of healing. In 21 patients (group 2) of our study the fracture was treated non-arthroscopically, but diagnostic arthroscopy was conducted 6.7 ± 3.5 months thereafter and glenohumeral lesions were found in 6 patients (28.5%). Interestingly, this result was similar to patients of group 1 (25%) which shows that lesions may not dissolve over time. In contrast, not all patients that suffered from a glenohumeral lesion were symptomatic at that time. However, 2 patients were symptomatic and diagnostic arthroscopy revealed a full thickness rotator cuff tear. While both patients were asymptomatic before the fracture event there is little to judge, whether this cuff tear was a result of the trauma, or not. Nevertheless, Tempelhof et al. proclaim that there are certain parameters that may convent an asymptomatic rotator cuff tear into a symptomatic tear, however, for a fracture of the distal clavicle this remains speculative [16]. Symptoms may have been misinterpreted as unspecific subacromial impingement caused by the implant itself. One may argue that symptoms may have resolved with hardware removal alone.

There are several limitations of this study: First, the number of patients is low and the results may be biased due to a retrospective design. Secondly, patients were excluded if the fracture was not classified as Neer type II. Thus, this study does not evaluate the total amount of concomitant glenohumeral lesions in distal clavicle fractures. In fact, there may have be a selection bias because of the indication for an arthroscopically assisted approach with coracoclavicular ligament augmentation. However, we suggest that a Neer type IIb fracture represents a distal clavicle fracture with extensive tissue damage, to be distinguished from the more mild type I injuries, according to Neer himself [16]. During the period of study both implant types have been applied. In the early phase hook plate system dominated, later subsequently locked plating and arthroscopically assisted coracoclavicular fixation has become the method of choice. The study period implies a development process of the implants. For this reason we were unable to randomize and thus study is retrospective. Our preliminary and already published work regarding the used implants has demonstrated a tendency better functional outcome after the arthroscopically assisted osteosynthesis [17]. Therefore this treatment has become to our method of choice. The tendency of better functional outcome was further strengthened by this study.

There is no recommendation such fractures to be referred only by arthroscopic experienced surgeons. However the awareness in case of persistent complaints either to perform an arthroscopically assisted implant removal or at least MRI-Imaging after implant removal is necessary. This treatment phase is not that critical of the time; priming of arthroscopically assisted implant removal should be possible.

## Conclusion

Concomitant intra-articular glenohumeral injuries in type Neer II distal clavicle fractures are not only observed during fracture treatment, but also 7 months thereafter in more than a quarter of cases. As glenohumeral lesions may not dissolve over time, diagnostic arthroscopy is recommended in cases were hardware removal is indicated and the patient is symptomatic. In turn, early diagnosis and treatment of relevant concomitant intra-articular glenohumeral injuries is reasonable, as delayed diagnosis and treatments might delay recovery. Whether or not glenohumeral pathologies concomitant to a distal clavicle fracture are of traumatic origin, however, remains uncertain.

## Abbreviations

AC: Acromioclavicular
CS: Constant score
HAGL: Posterior avulsion of the glenohumeral ligament
IGHL: Inferior gleno-humeral ligament
LCP: Locking compression plate
LMU: Ludwig Maximilians University
MGHL: Medial gleno-humeral ligament
MRI: Magnetic Resonance Imaging
OSS: Oxford shoulder score
SGHL: Superior glenohumeral ligament
SLAP: Superior labrum anterior-posterior complex
